# A peptide-DNA hybrid bio-nanomicelle and its application for detection of caspase-3 activity

**DOI:** 10.3389/fchem.2022.1005315

**Published:** 2022-09-06

**Authors:** Fan Zhang, Changqing Mao, Siyu Cao, Runchi Zhang, Yi Guo, Guifang Chen, Chang Feng

**Affiliations:** Center for Molecular Recognition and Biosensing, School of Life Sciences, Shanghai University, Shanghai, China

**Keywords:** peptide-DNA hybrids, bio-nanomicelle, caspase-3, activity detection, cell lysates

## Abstract

Bio-nanomicelles based on biomaterials such as nucleic acids, peptides, glycans, and lipids have developed rapidly in the field of bioanalysis. Although DNA and peptides have unique advantages, unfortunately, there are few bio-nanomicelles integrating DNA with peptides. Here, we designed a peptide-DNA hybrid bio-nanomicelle for the activity detection of caspase-3. The detection mechanism is based on caspase-3 specific recognition and cleavage of peptide substrates, which owns high sensitivity and selectivity. Under optimal conditions, the detection of caspase-3 activity can be achieved using our designed bio-nanomicelles and the detection limit is 0.72 nM. Furthermore, the proposed method was also successfully applied for the detection of caspase-3 in cell lysate samples after apoptosis-inducing.

## Introduction

Nanomicelles are supramolecular nanostructures formed by self-assembly in solution of amphiphilic copolymers with hydrophilic and hydrophobic groups, including block polymers (Peeler et al., 2019; Shah and Leon, 2019; Yang et al., 2020) and graft polymers (Yuan et al., 2019). The micelles have a core-shell structure with a hydrophobic core that can be loaded with hydrophobic molecules and a hydrophilic exterior that allows them to be stabilized in aqueous media. For example, encapsulation of fluorescent 1-pyrenecarboxaldehyde (Py-CHO) molecules into block copolymer-based micelles not only provides a hydrophobic environment for the accommodation of Py-CHO, but also enhances its fluorescence properties (Gao et al., 2018). The excellent stability of micelles and the ideal encapsulation and protection of hydrophobic probes (Cheng et al., 2021; Liang et al., 2021) have received much attention in the field of biochemical analysis.

In recent years, bio-nanomicelles based on biomaterials such as nucleic acids, peptides, glycans, and lipids have developed rapidly in the field of bioanalysis. Compared with other polymeric micelles, DNA micelles are easy to prepare, small in size (<100 nm), and own supramolecular structure with precisely controllable nucleotide, which have great potential in detecting cancer cells and designing other innovative structures (Wang et al., 2016; Jin et al., 2017). Peptides can also be used for micelle construction, where peptides are formed into peptide nanomicelles by designing the sequence, degree of polymerization, and intermolecular interactions, i.e., hydrogen bonds, π-π, hydrophobic interactions, etc., which not only show high stability but also have several biomedical effects, including long-term cycling, enzymatic cleavage properties, etc. (Callmann et al., 2020; Shuai et al., 2020). In addition, both DNA and peptides can be coupled with signaling molecules (e.g. fluorescent groups and MRI contrast agents) that can be used for fluorescence detection and imaging, etc (Hill et al., 2019; Park et al., 2020; Schnurr et al., 2020). Although both materials have unique advantages, unfortunately, there are few bio-nanomicelles integrating DNA with peptides. If the advantages of the two materials can be combined to construct micelles and perform specific functions, it will be important to promote the development and application of bio-nanomicelles.

Caspases are a family of cysteine proteases that play an important role in the apoptotic pathway (McComb et al., 2019). Apoptosis is of great significance to organisms. Abnormal apoptosis processes can lead to disease states of life, such as tumors, neurological disorders, and fibrosis diseases (Shalini et al., 2015; Hinz and Lagares, 2019; Pham et al., 2020). Caspase-3 is an important protein in this family that mediates the apoptotic pathway and is considered a biomarker of apoptosis (Grütter, 2000). At present, several methods have been developed for the detection of caspase-3 activity, including colorimetry, fluorescence and electrochemical method, *etc*. For example, the method based on gold nanoparticles (AuNPs) allows directly observe the test results with the naked eye, but it is time-consuming and has low sensitivity (Pan et al., 2012). The construction of fluorescent probe technology based the principle of fluorescence resonance energy transfer (FRET) is simple, but its sensitivity and applicability are limited due to the absence of signal amplification strategy (Li et al., 2015). Although the electrochemical method has high sensitivity, it requires a complex chemical modification process, which causes instability factors for the detection system (Song et al., 2018; Shin et al., 2022). Therefore, it is necessary to develop simple and rapid methods for sensitive and specific analysis of caspase-3 activities.

**SCHEME 1:**
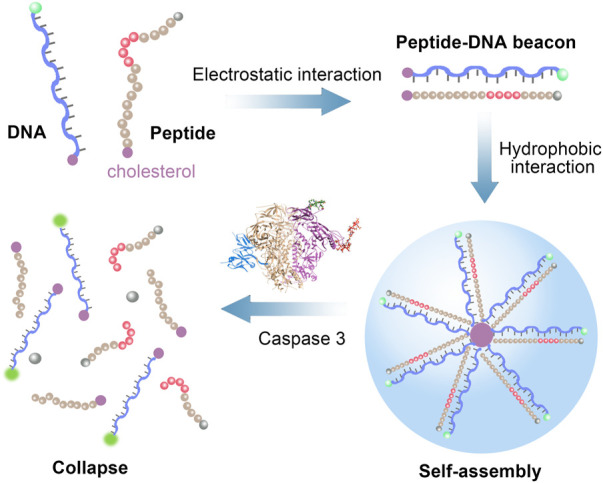
Schematic illustration of peptide-DNA hybrid bio-nanomicelles for caspase-3 detection.

In our work, we designed a peptide-DNA hybrid bio-nanomicelles for the detection of caspase-3 activity (Scheme 1). Cholesterol-modified DNA and peptides are mixed in a certain proportion. Since DNA is negatively charged and the peptide is positively charged, they can bind to each other by the electrostatic interaction. At the same time, under the hydrophobic function of cholesterol, DNA and peptide will assemble into ordered nanomicelles of a certain size. *In vitro* experiments have demonstrated that peptide-DNA hybrid bio-nanomicelles can be formed stably and specifically recognize and cleave the C-terminus of peptide Asp-Glu-Val-Asp (DEVD) when caspase-3 is present (Deng et al., 2019). Then, fluorescent measurements are conducted for the assay of caspase-3 activity by releasing the fluorophore at the ends of DNA. With this strategy, caspase-3 activity has been successfully detected using our designed peptide-DNA hybrid bio-nanomicelles with high sensitivity and specificity.

## Material and methods

### Materials and reagents

All oligonucleotides used in this research Supplementary Table S1 were synthesized by Sangon Biotech. Co., Ltd. (Shanghai, China) with HPLC purification. Peptide was synthesized and purified by Shanghai Scipeptide Co., Ltd. (Shanghai). Other chemical reagents were all of analytical grade. All solutions were prepared with Milli-Q water (18.2 MΩ cm^−1^) purified with a Milli-Q purification system (Millipore, USA). Nile Red, Z-DEVD-FMK and lysozyme were purchased from Shanghai Aladdin Bio-Chem Technology Co., LTD. Caspase-1, caspase-3 and trypsin were purchased from Abcam Trading Co., Ltd. (Shanghai). Bovine serum albumin (BSA) and BCA protein determination kit were purchased from Beijing Solarbio Science & Technology Co., Ltd. Commercial Caspase-3 Activity Detection Kit was purchased from Beyotime Biotechnology. Ultrafiltration centrifuge tubes were purchased from Millipore (Shanghai) Trading Co., Ltd.

### Construction of peptide-DNA hybrid bio-nanomicelles

The peptide-DNA hybrid bio-nanomicelles were prepared by mixing peptide and DNA in a certain proportion in PBS buffer (137 mM NaCl, 2.7 mM KCl, 10 mM Na_2_HPO_4_, and 2 mM KH_2_PO_4_, pH 7.4) and sonicated for 2 h at 25 °C.

### Atomic force microscope (AFM) and ζ-potential characterization of peptide-DNA hybrid bio-nanomicelles

Peptide-DNA hybrid bio-nanomicelles were prepared according to the synthesis method in the previous step. Then 20 μL of above samples were scanned according to the operating instructions for *ex situ* Agilent 5500 AFM system (Agilent Technologies, US). The sample was dropped on the mica slice, and then dried with nitrogen gas. The scanning speed was 0.3 Hz and resonant frequency of AFM tips was 160–260 kHz. Morphological analysis of the peptide-DNA hybrid bio-nanomicelles was obtained at a resolution of 512 × 512 pixels. The ζ-potential of the nanomicelles (600 nM, in PBS buffer) was measured by Zetasizer nano-ZS (Malvern, United Kingdom) at 25°C.

### Fluorescence detection

The formation of hybrid nanomicelles was analyzed using a F-7000 fluorescence spectrometer (Hitachi High-Technologies, Japan). The main parameters of the instrument were set to a slit width of 5 nm and an excitation light voltage of 750 V. FAM-DNA was excited at 492 nm and the spectra were measured in the range from 500 to 600 nm.

### Determination of critical micelle formation concentration

The CMC of hybrid nanomicelles was determined by fluorescent Nile red encapsulation assay. 100 μM of Nile red was incubated with a series of assembly concentrations of bio-nanomicelle samples (Range from 0 to 1,500 nM) in 50 μL of PBS buffer. The samples were heated to 90 °C for 5 min and slowly cooled to 37 °C. After incubation at 37 °C for another 1 h, the fluorescence intensity of Nile red was recorded in the range from 560 to 760 nm with an excitation wavelength of 535 nm. The CMC could be calculated by tracking the relationship between fluorescence intensity of Nile Red and function of the sample concentration.

### Nanoparticle tracking analyzer characterization of peptide-DNA hybrid bio-nanomicelles after caspase-3 digestion

After incubation with 10 μl of 50 nM caspase-3 at 37°C for 1 h, the particle concentration and size distribution of bio-nanomicelles was analyzed by using a NanoSight LM (Malvern, United Kingdom). The samples were injected in the sample chamber with sterile syringes until the liquid reached the tip of the nozzle. All measurements were performed at room temperature.

### *In vitro* detection of caspase-3 activities

In the experiments of quantitative detection of the enzyme activity, different concentrations of caspase-3 were incubated with 600 nM bio-nanomicelles at 37 °C for 1 h. The fluorescence intensity was measured by using a F-7000 fluorescence spectrometer. As for the determination of caspase-3 inhibitor, different concentrations of Z-DEVD-FMK were mixed with 50 nM caspase-3 at room temperature for 30 min. Subsequently, 600 nM bio-nanomicelles were incubated with the mixture at 37°C for 1 h followed by fluorescent detection.

### Cell culture and apoptosis induction assay

HeLa cells (Human cervical cancer cells) were obtained from the cell bank of the Cell Bank of the Committee on Type Culture Collection of the Chinese Academy of Sciences. The cells were cultured in DMEM medium containing 1% penicillin-streptomycin and 10% FBS at 37 °C in humidified air containing 5% CO_2_. These cells are selected at the end of the log phase and cell numbers were determined with cell counter (Biorad, United States). HeLa cells (2 × 10^5^ cells/well) were pre-cultured in a 6-well plate and incubated with 1 ml apoptosis inducer for 0, 4, 8, 12 h, respectively. HeLa cells without apoptosis inducer were used as the control.

### Detection of caspase-3 activities in cell lysates

The cells were collected using a scraper and washed with PBS buffer twice. 500 μL lysis buffer was added into the cell residues and placed on ice for 30 min. After centrifugation, the supernatant was collected as HeLa cell lysate. A BCA protein assay kit was used to determine total protein of each lysate. For cellular caspase-3 activity tests, 500 μl cell lysate was incubated with 600 nM bio-nanomicelles at 37°C for 1 h. In addition, the activities of caspase-3 in the HeLa cell lysate were in parallel measured with a commercial kit.

## Results and discussion

### Construction of peptide-DNA hybrid bio-nanomicelles

**FIGURE 1:**
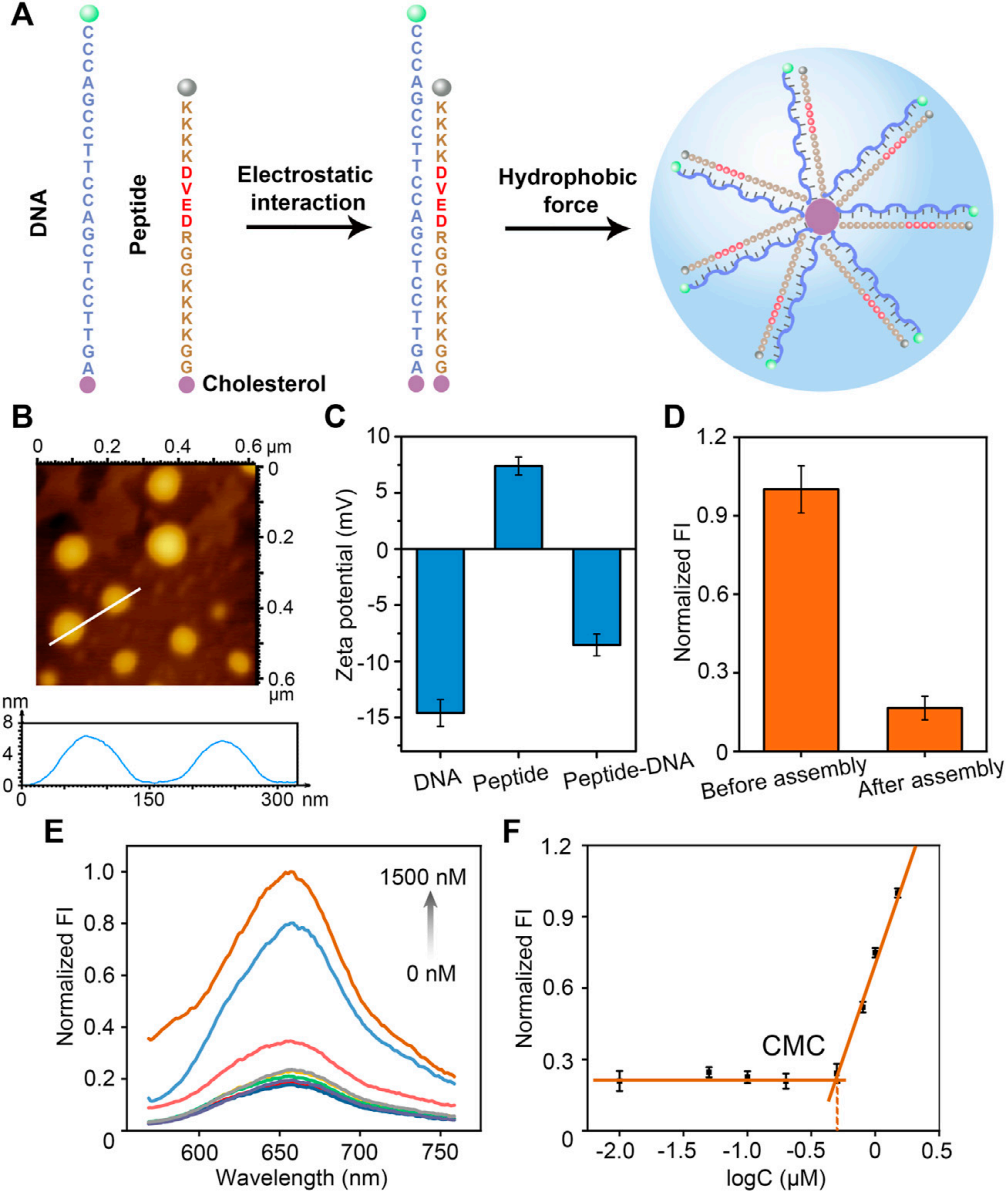
Design, construction and characterization of peptide-DNA hybrid bio-nanomicelles. **(A)** Sequence design and formation principle of peptide-DNA hybrid bio-nanomicelles (Caspase-3 specific recognition cleavage sites in red) **(B)** AFM characterization of peptide-DNA hybrid bio-nanomicelles **(C)** ζ-Potential characterization of peptide-DNA hybrid bio-nanomicelles before and after assembly. **(D)** Fluorescence characterization of peptide-DNA hybrid bio-nanomicelles before and after assembly. Excitation and emission wavelength of FAM is 492 and 522 nm. **(E)** and **(F)** Nile red encapsulation method to determine the CMC of peptide-DNA hybrid bio-nanomicelles. Excitation and emission wavelength of Nile red is 535 and 655 nm.

It is well known that DNA is negatively charged due to its phosphate backbone. We designed the peptide sequence to make the peptide positively charged. The polypeptide sequence is GGKKKKGGRDEVDKKKK, where Lys (K) and Arg (R) are positively charged and Asp (D) and Glu (E) are negatively charged, and overall the polypeptide is positively charged. Therefore, negatively charged DNA and positively charged polypeptide can bind together due to electrostatic adsorption. Moreover, the end of DNA and polypeptide are modified with cholesterol, the hydrophobic effect of which makes the polypeptide-DNA orderly self-assemble into a certain size of nanomicelles (Figure 1A). The formed peptide-DNA hybrid bio-nanomicelles were characterized by AFM and it was observed that the nanomicelles were spherical in shape with a size of about 150 nm (Figure 1B). Since DNA is negatively charged and peptide is positively charged, the electronegativity of the two diminishes upon binding, which was confirmed by ζ-potential characterization (Figure 1C). In addition, FAM fluorophore is modified with the 5′-terminal strand of DNA and BHQ quencher is modified with the other end of peptide, respectively. Supplementary Figure S1 showed comparison of the fluorescence intensity of peptide-DNA hybrid bio-nanomicelles at different concentration ratios. The fluorescence intensity increased gradually as the ratio of DNA in micelles increasing, indicating that equal amounts of peptides and DNA can ensure assembly of hybrid nanomicelles when FAM and BHQ are in close proximity and the fluorescent signal of FAM is quenched. Significant decrease of fluorescence intensity was found after assembly of DNA and peptide, indicating that DNA-FAM and peptide-BHQ successfully bound together (Figure 1D).

The critical micelle formation concentration (CMC) is critical for the construction of micelles. CMC of peptide-DNA hybridized micelles was calculated by the Nile Red assay as previously reported (Yin et al., 2019). Nile Red showed very weak fluorescence in a hydrophilic environment and strong fluorescence in a hydrophobic environment. Therefore, the CMC can be determined by measuring the fluorescence intensity of Nile Red when co-incubated with a series of diluted peptide-DNA hybrid micelle solutions (Figure 1E). The relationship between fluorescence intensity of Nile Red and function of the sample concentration was performed to estimate the value of CMC. When the concentration is lower than CMC, the hydrophobic interaction of cholesterol molecules is insufficient to lead to formation of micelle, and the fluorescence intensity of Nile Red is almost constant. However, once the concentration exceeds CMC, Nile Red is encapsulated in the micelle core and the fluorescence increases dramatically. Therefore, the intersection of the tangent line of the intensity ratio with the horizontal line of relatively constant value can be estimated as the value of CMC (500 nM) (Figure 1F). To ensure successful micelle formation, all hybrid peptide-DNA nanomicelles in this study were assembled at concentrations higher than 500 nM. The results above indicate that DNA and peptides can successfully assemble and form bio-nanomicelles of a certain size.

### Validation of caspase-3 digestion

**FIGURE 2:**
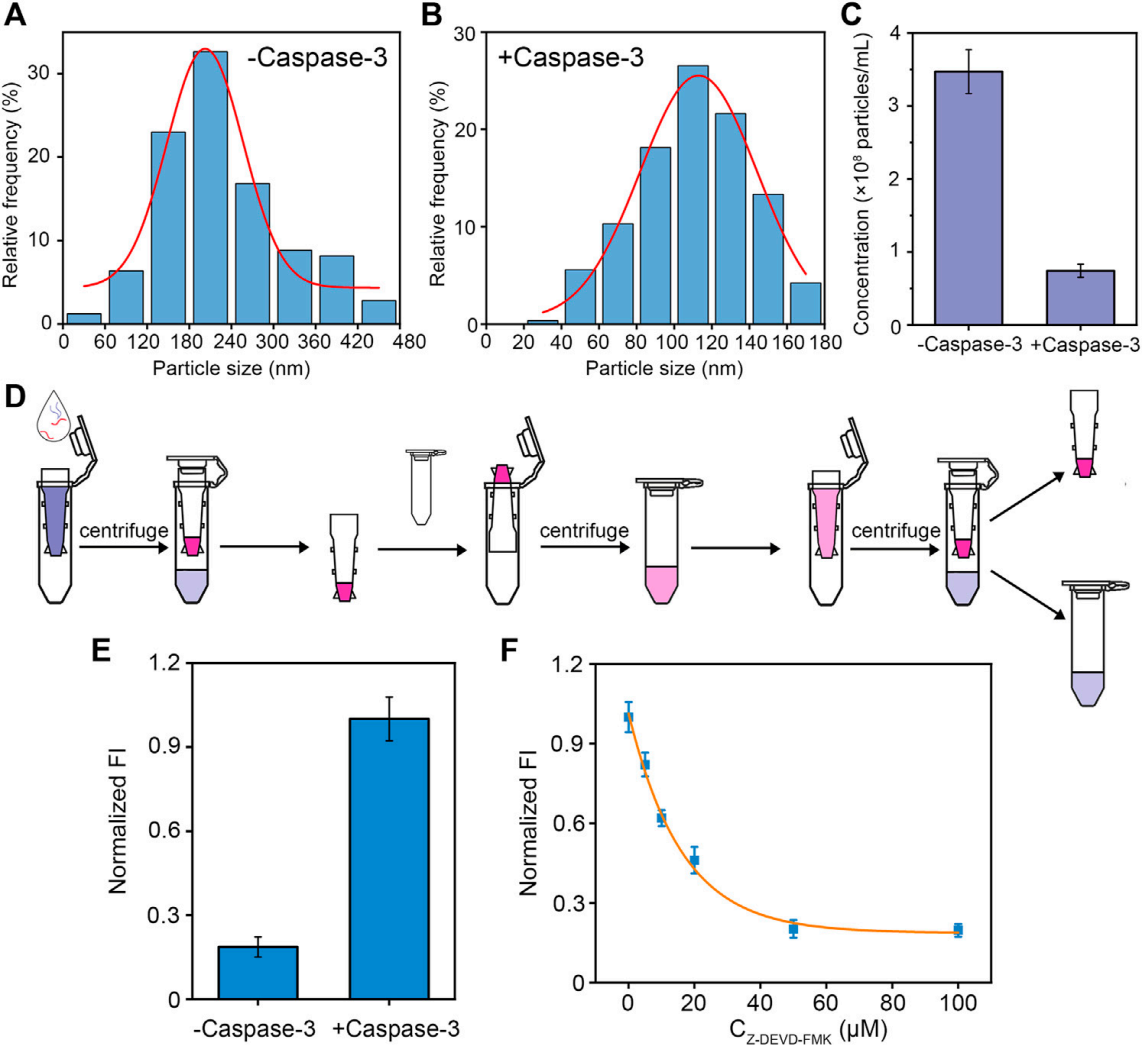
Validation of caspase-3 digestion. **(A,B)** NTA characterization of particle size distribution of bio-nanomicelles assembled. Before and after digestion of caspase-3. **(C)** NTA characterization of particle concentration of bio-nanomicelles before and after digestion of caspase-3. **(D)** Schematic diagram of detecting fluorescence intensity of bio-nanomicelles before and after digestion of caspase-3. **(E)** Fluorescent detection of bio-nanomicelles before and after digestion of caspase-3. Excitation and emission wavelength of FAM is 492 and 522 nm. **(F)** Relationship between inhibitors of different concentrations of caspase-3 and fluorescence intensity of bio-nanomicelles.

**FIGURE 3:**
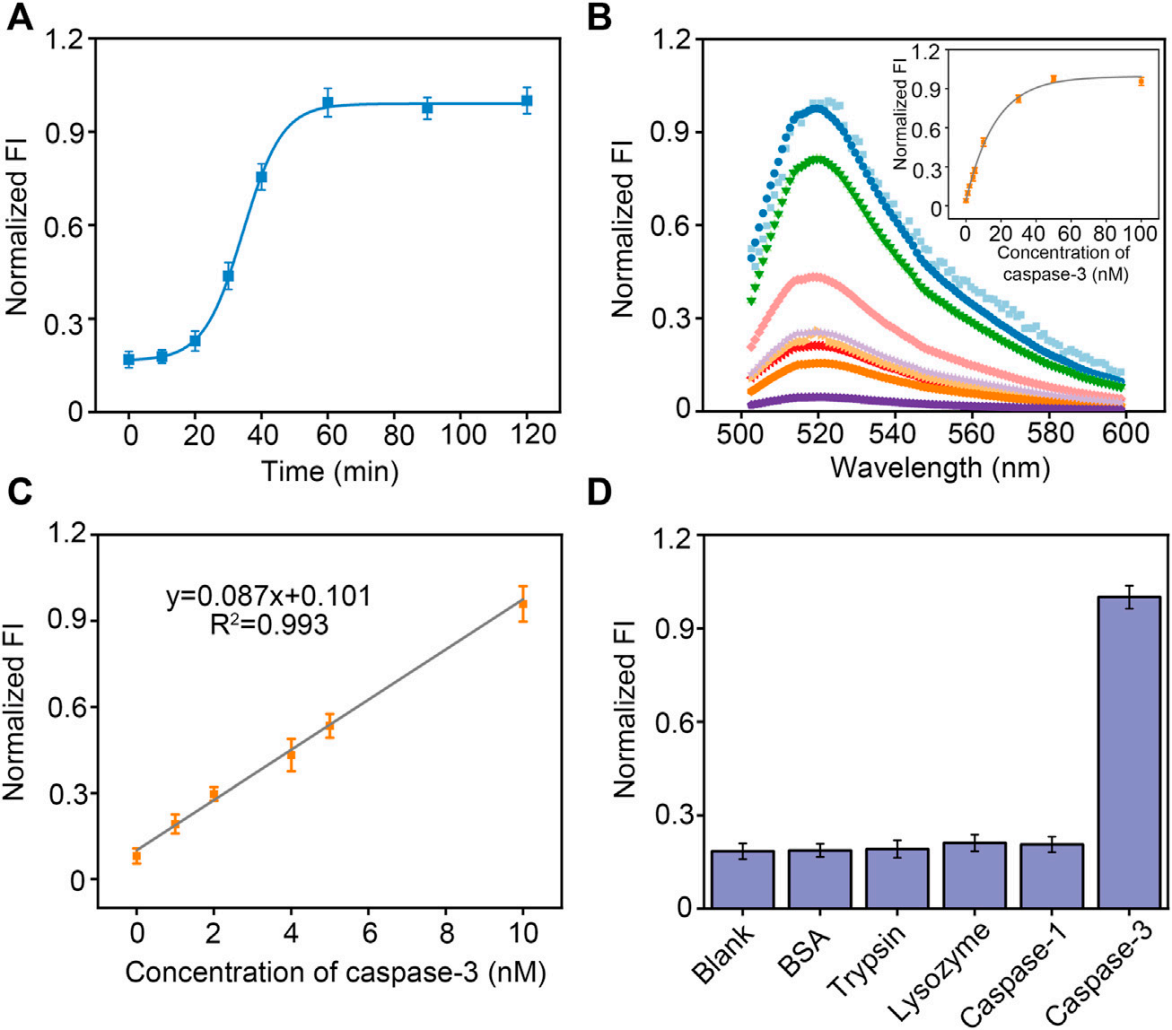
**(A)** Kinetic analysis for the caspase-3 using peptide-DNA hybrid bio-nanomicelles. **(B)** Fluorescence intensity curve with concentration of caspase-3. **(C)** Linearity of fluorescence intensity with concentration of caspase-3. **(D)** Comparison of fluorescence intensity in the presence of different protein and proteases. Excitation and emission wavelength of FAM is 492 and 522 nm.

To demonstrate the effectiveness of our designed peptide-DNA hybrid bio-nanomicelles for caspase-3 detection, NTA and fluorescent assay were used to validate the digestion of caspase-3. As shown in Figures 2A,B, the successfully assembled bio-nanomicelles had a particle size of approximately 180 nm and the particle concentration decreased significantly after the addition of caspase-3, indicating that the addition of caspase-3 cleaves the peptide and disassembles the bio-nanomicelles. It can also be seen from the NTA video screenshot in Supplementary Figure S2 that the amounts of bio-nanomicelles are significantly reduced after the addition of caspase-3. Statistical analysis of the concentration of bio-nanomicelles before and after the addition of caspase-3 revealed that the concentration decreased about 6 times after digestion of caspase-3 on bio-nanomicelles (Figure 2C). Figure 2D shows the flow chart to detect the fluorescence intensity before and after assembly. After the addition of caspase-3, the peptide is specifically cleaved by caspase-3, the BHQ quencher at the end of the peptide released, and the fluorescence of FAM at the end of the DNA restored, generating significant fluorescent signal (Figure 2E). To demonstrate that the disassembly of bio-nanomicelles is due to the catalytic activity of caspase-3, different concentrations of caspase-3 inhibitor (Z-DEVD-FMK) were incubated with samples containing 50 nM caspase-3 for 30 min prior to the assay. As seen in Figure 3F, the fluorescence intensity decreased with increasing concentration of caspase-3 inhibitor. The signal response of the sample containing the inhibitor was much lower than that in the absence of the inhibitor, indicating that caspase-3 activity plays an important role in the disintegration of bio-nanomicelles. The IC_50_ value (defined as 50% inhibition efficiency) of Z-DEVD-FMK against caspase-3 was determined to be 20 μM. The result also show that our method has potential as a useful tool in the screening of protease inhibitors.

### Detection performance of peptide-DNA hybrid bio-nanomicelles

We next monitored caspase-3 activity dynamically in real time using bio-nanomicelles. Once 10 nM caspase-3 was added, the fluorescence emission at 520 nm was measured continuously from 0 min onwards. Figure 3A depicts a typical real-time fluorescence curve, showing that the fluorescence intensity increases slowly and then sharply from 20 min and almost reaches saturation after 1 h. We speculate that the cleavage of the peptide by caspase-3 leads to a slow increase in fluorescence intensity followed by a sharp increase because more and more peptides are cleaved and the bio-nanomicelles are electrostatically unbalanced, leading to micellar disintegration. This result confirms that our bio-nanomicelles are feasible for continuous monitoring of caspase-3 activity. The fluorescence intensity of the samples increased with increasing concentration of caspase-3 (Figure 3B) and had a good linear relationship from 1–10 nM. The detection limit is 0.72 nM (LOD = 3σ/slope) (Figure 3C). In addition, specific detection of caspase-3 using bio-nanomicelle was assessed by adopting other interfering proteins or proteases, including BSA, lysozyme, trypsin and caspase-1. Even when the interfering protein was 20 times the concentration of caspase-3, the reaction system did not show any significant fluorescence activation (Figure 3D), validating the excellent selectivity of our method.

### Application in apoptosis induced HeLa cell lysates

**FIGURE 4:**
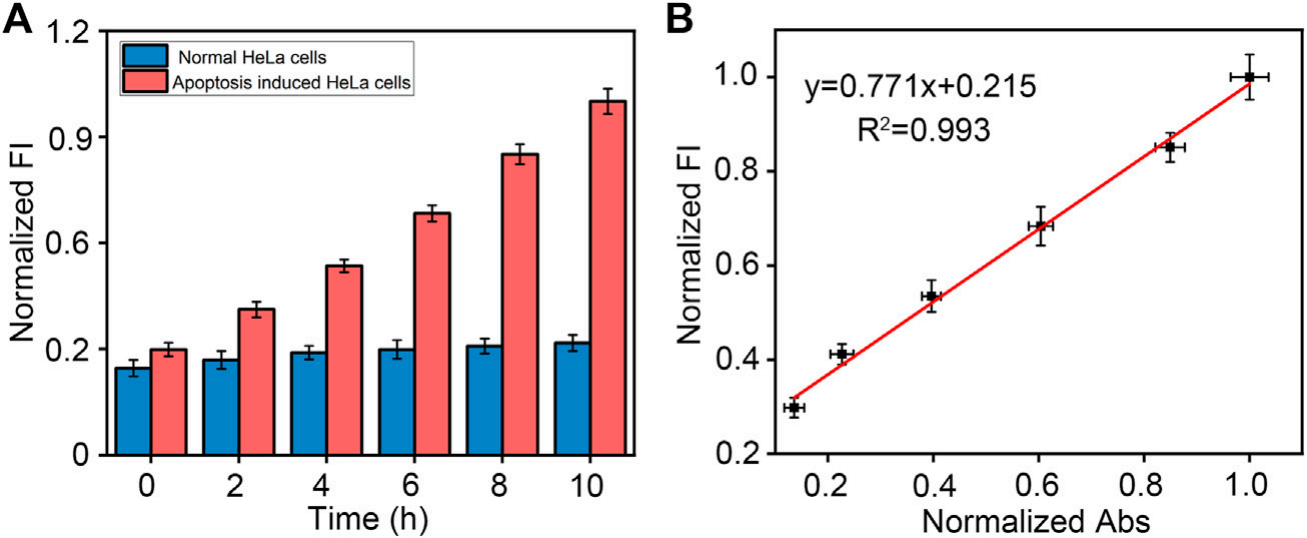
**(A)** Detection of caspase-3 in HeLa cell lysates with or without apoptosis-inducing over time using our designed method. Excitation and emission wavelength of FAM is 492 and 522 nm. **(B)** Comparison of commercially available caspase-3 assay kits with our designed method.

Finally, due to the important role caspase-3 plays in apoptosis, we investigated the application of peptide-DNA hybrid bio-nanomicelles. In complex cell lysates. For detecting different concentrations of caspase-3, our method also has good reproducibility and recovery (Table 1). Real cell apoptosis assays were also conducted to validate the performance of our method. As shown in Figure 4A, the longer the apoptosis inducer was incubated with the cells, the higher the fluorescence intensity of the cell lysate at 520 nm, which indicated that more active caspase-3 was produced after induction. In contrast, control experiments performed with HeLa cells in the absence of inducer did not show any significant increase in fluorescence intensity upon incubation. A commercially available caspase-3 cell activity assay kit was also used and confirmed the presence of active caspase-3 in apoptosis-induced HeLa cell lysates (Supplementary Figure S3), with a good linear correlation between fluorescence intensity and absorbance compared to our method, demonstrating the reliability and validity of our assay (Figure 4B). These results suggest that this method can be used to detect caspase-3 activity during apoptosis.

**TABLE 1:** Determination of Caspase-3 in the Cell Extract Sample Using Our Designed bio-nanomicelles.

Sample	Caspase-3 spiked (nM)	Caspase-3 measured (nM)	Recovery (%)	RSD (%) (n = 3)
1	0	0.894		4.8
2	1.148	2.131	107.73	3.2
3	5.002	5.564	93.37	4.7

## Conclusion

In summary, we have developed a peptide-DNA hybrid nanomicelles-based fluorescence assay for detection of caspase-3 activity. This fluorescence strategy is mainly achieved by construction of peptide-DNA hybrid bio-nanomicelles and caspase-3 specific recognition and cleavage of peptide substrates, The method can be used to detect caspase-3 activity and its inhibitor, and has been successfully applied to the detection of caspase-3 activity in cell lysate samples after apoptosis-inducing. In our assay, we combined the advantages of precise control of DNA and biomedical effects of peptides to construct hybrid micelles, which expanded the application scope of biological nanomicelles. The three-dimensional nanomicelle fluorescent probe has the dual functions of specific recognition and signal amplification, which could achieve sensitive and specific detection of caspase-3 activity. In addition, the synthesis process of biological nano-micelles is simple, and the synthetic material is environmentally friendly, owning good stability and biocompatibility. Furthermore, adopting suitable peptide sequences or nucleic acid sequences instead of DEVD, other proteases or nucleases can also be assayed using our designed peptide-DNA hybrid nanomicelles.

## Data Availability

The original contributions presented in the study are included in the article/Supplementary Material, further inquiries can be directed to the corresponding authors.
