# Supplementary material for: A peptide-DNA hybrid bio-nanomicelle and its application for detection of caspase-3 activity
Source: Front Chem. 2022 Sep 6;10:1005315. doi: 10.3389/fchem.2022.1005315 (PMC9485609; doi:10.3389/fchem.2022.1005315)
Supplement: Supplementary file 1 [file DataSheet1.pdf]

Supplementary Material

1 Supplementary Figures and Tables

1.1 Supplementary Tables

Supplementary Table 1. Sequences of oligonucleotide and peptide used in this work.

Name	Sequences	Modification
DNA	CCCAGCCTTCCAGCTCCTTGA	Cholesterol, 5'-FAM
Peptide	GGKKKKGGR*DEVD*KKKK	Cholesterol, BHQ1

The italics indicates the cleavage sites of caspase-3.

1.2 Supplementary Figures

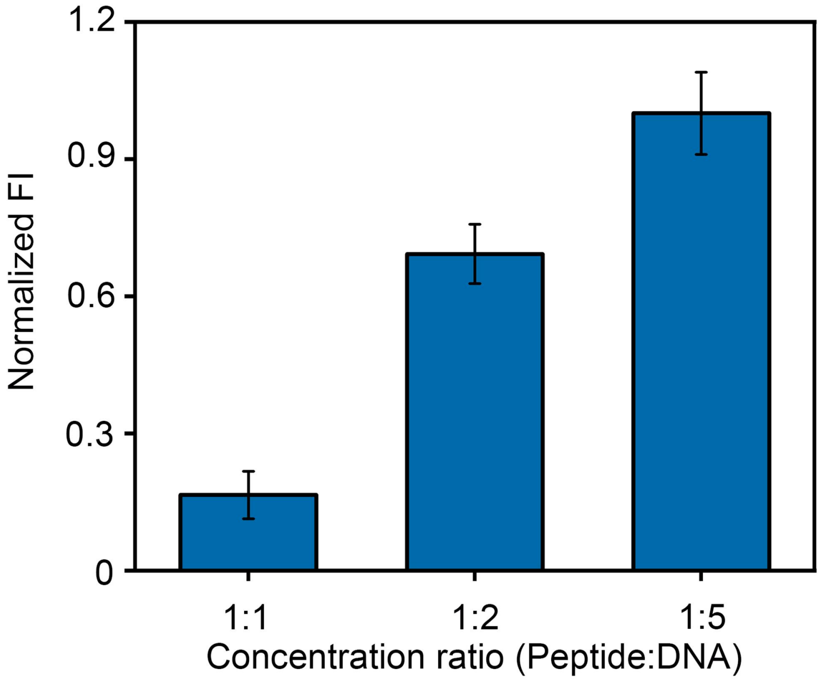

Supplementary Figure 1. Comparison of the fluorescence intensity of peptide-DNA hybrid bio-nanomicelles at different concentration ratios.

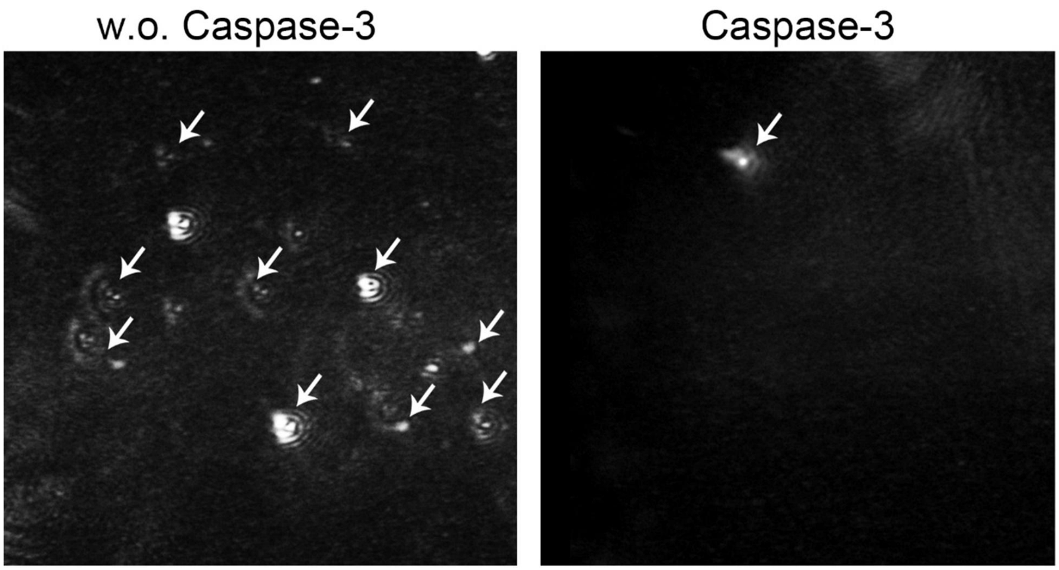

Supplementary Figure 2. NTA video screenshots of peptide-DNA hybrid bio-nanomicelles without or with digestion of caspase-3.

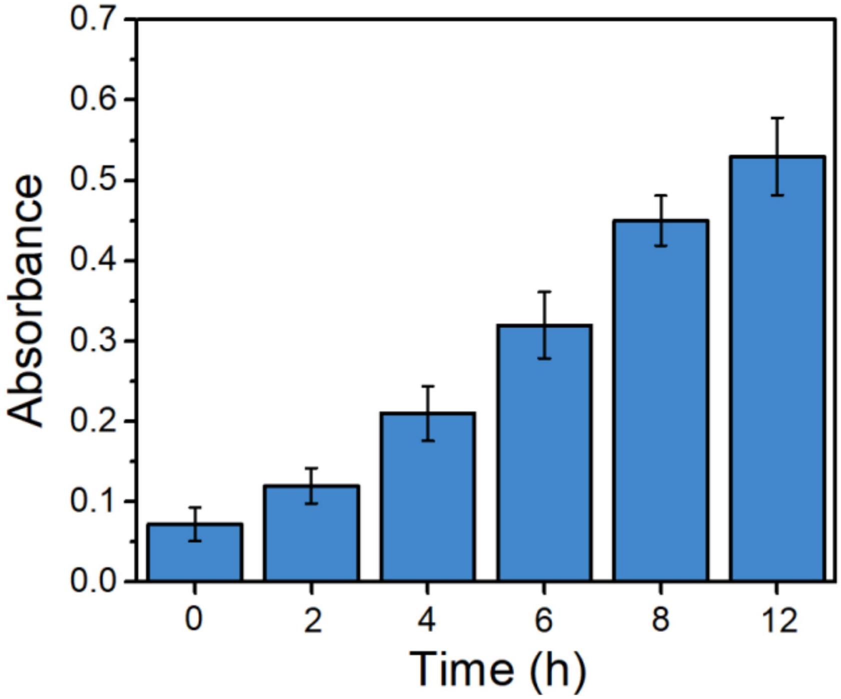

Supplementary Figure 3. Commercial caspase-3 apoptosis kit to detect apoptosis-induced HeLa cells.
